# Imaging Review of Skeletal Tumors of the Pelvis—Part I: Benign Tumors of the Pelvis

**DOI:** 10.1100/2012/290930

**Published:** 2012-05-15

**Authors:** Gandikota Girish, Karen Finlay, Yoav Morag, Catherine Brandon, Jon Jacobson, David Jamadar

**Affiliations:** ^1^Department of Radiology, University of Michigan, 1500 E. Medical Center Drive, TC-2910, Ann Arbor, MI 48109-0326, USA; ^2^Department of Radiology, Henderson Hospital, Hamilton Health Sciences and McMaster University, 711 Concession Street E, Hamilton, ON, Canada L8V 1C3

## Abstract

The osseous pelvis is a well-recognized site of origin of numerous primary and secondary musculoskeletal tumors. The radiologic evaluation of a pelvic lesion often begins with the plain film and proceeds to computed tomography (CT), or magnetic resonance imaging (MRI) and possibly biopsy. Each of these modalities, with inherent advantages and disadvantages, has a role in the workup of pelvic osseous masses. Clinical history and imaging characteristics can significantly narrow the broad differential diagnosis for osseous pelvic lesions. The purpose of this review is to familiarize the radiologist with the presentation and appearance of some of the common benign neoplasms of the osseous pelvis and share our experience and approach in diagnosing these lesions.

## 1. Introduction

The bony pelvis is a well-recognized site of origin of numerous primary and secondary musculoskeletal tumors. In adulthood, the pelvis is one of the areas of the axial skeleton where haematopoietic red marrow predominates. The specific radiological diagnosis of primary pelvic tumors is often difficult, due to their varied appearance and overlapping radiological features. The aim of this review is to familiarize radiologists with the multimodality appearances of benign neoplasms of the bony pelvis.

**Figure 1:**
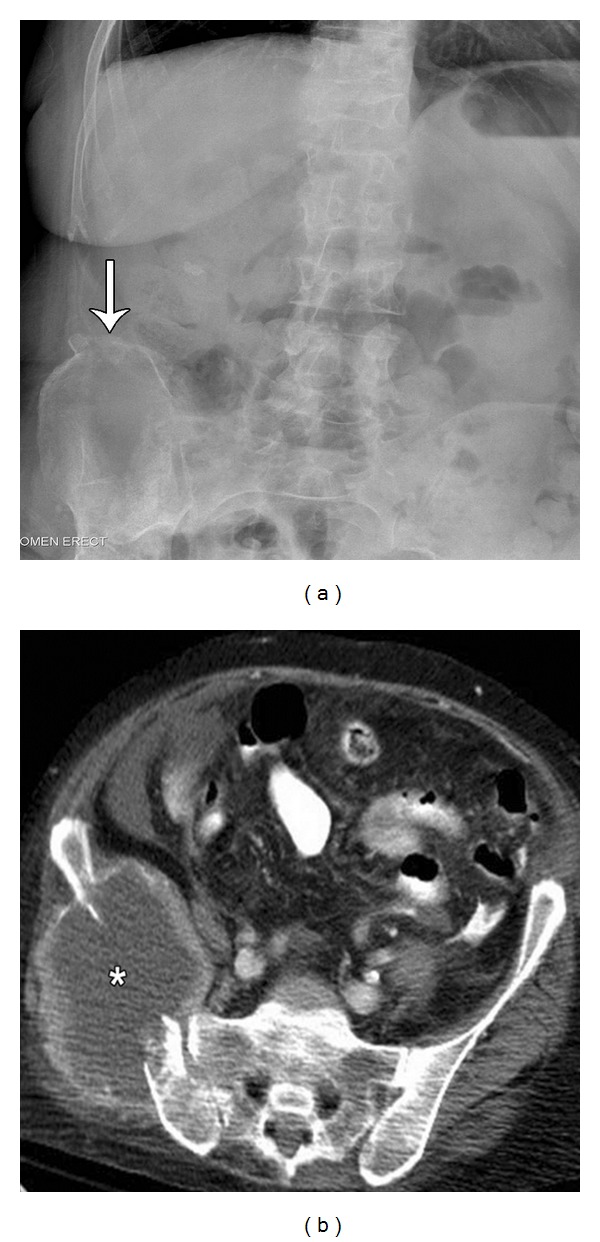
Middle-aged female presents with chronic pelvic pain. Frontal radiograph (a) of the abdomen reveals subtle cortical disruption and periosteal reaction involving the superior aspect of the right iliac crest (arrow). Subsequent CT examination (b) demonstrates a lytic destructive lesion of the right iliac bone, with a large soft tissue mass (asterisk).

One of the main challenges in diagnosing tumors of the pelvis lies in the low sensitivity of plain radiographic detection (Figures 1(a), and 1(b)). Unless there is gross cortical destruction, extensive periosteal reaction or a large soft tissue component, with or without calcification, pelvic tumors are often not obvious on plain radiographs. Plain radiography is the initial screening modality for most symptomatic patients; thus, a proper history including age, rate of onset, and duration of symptoms is necessary to raise the level of suspicion. In addition, a pelvic tumor is often obscured on the frontal projection, further complicated by overlying bowel gas. There should therefore be a low threshold for requesting multiple views (inlet view, outlet view, judet view, etc.) of the pelvis. Often these lesions are best visualized on non-AP radiographs. Assessment of pelvic bones should be an integral part of the reporting process of all abdominal films.

## 2. Imaging Techniques

Multimodality imaging and a combined multidisciplinary approach (radiologist, orthopedic surgeon, pathologist, and primary care physician) is essential in accurate assessment and staging of the tumor involved and facilitation of good patient care. While the innovative technology of cross-sectional imaging is useful for accurate staging and defining the local extent of the disease, the plain film appearance is most helpful in providing the differential diagnosis. Debate exists as to which modality, CT or MR, is better in characterizing the tumors. Unlike soft tissue tumors, where MR has a distinct advantage, MR and CT are often complimentary in the evaluation of primary bone tumors [1]. CT provides important information regarding tumor location, margins, presence or absence of osseous or chondroid calcification, cortical involvement, and associated periosteal reaction. MRI has proven superior in assessing intraarticular/intraosseous extension and the presence of hemorrhage and intratumoral necrosis [2, 3].

## 3. Recent Advances in Tumor Imaging

Dynamic-enhanced MRI may help in differentiation of reactive bone edema from tumor extension, in a malignant lesion [4]. Tumor tissue enhances on early dynamic images; whereas peritumoral reactive edema may not show early phase enhancement [5–7]. This technique has the potential to assist the surgeon in operative planning for resection of disease. Quantitative dynamic MR imaging can quantify the degree of tumor necrosis following preoperative chemotherapy. This is because active tumor enhances more rapidly than nonviable tumor and post-treatment change [8, 9]. Thereby, chemotherapy can be altered if the response is not adequate.

Diffusion-weighted imaging (DWI) is a technique based on the fact that viable tumor restricts the diffusion of water when compared to normal tissue and is depicted as an increase in signal intensity in diffusion-weighted sequences [8]. DWI can be helpful in differentiating benign from malignant vertebral compression fractures [10].

MR Spectroscopy can measure different metabolites that make up the tumors. Even though single voxel and multivoxel MR spectroscopy have demonstrated elevated Choline levels in many malignant tumors, it can also be elevated in some benign tumors including giant cell tumors [11, 12]. Hence, it is not specific enough in diagnosing malignant tumors.

### 3.1. Positron Emission Tomography/Computed Tomography (PET/CT)

Presently, PET/CT machines incorporate advanced multidetector CT technology, which enables diagnostic quality CT images to be obtained. PET/CT is helpful in differentiating benign lesions from the malignant ones, assessing treatment response, and in postoperative tumor surveillance as it is very sensitive to early tumor recurrence [13]. Fluorine-labelled fluorodeoxyglusose (FDG) is the most commonly used PET tracer. It is preferentially taken up by the cells, which demonstrate increase in cellular glycolysis, seen in most malignant bone tumors and in some benign conditions like arthritis and infection [14–16]. The degree of uptake of the FDG tracer by the tumor cells when compared to the normal soft tissue background is expressed in terms of standardised uptake value (SUV), a marker of metabolic activity of the tumor [17]. A study involving 52 bone tumors by Aoki et al. concluded that average SUV value for benign bone lesions to be 2.18 and for the malignant ones to be 4.34 [18]. Round blue cell tumors like Ewing's sarcoma and bone lymphoma showed the greatest FDG uptake followed by moderate uptake in osteosarcoma and low uptake in chondrosarcoma. Some benign bone lesions, which can be FDG avid, include giant cell tumors, aneurysmal bone cyst, osteoblastoma (likely because of the presence of significant amount of giant cells) chondroblastoma, and fibrous dysplasia [18, 19]. Most benign bone lesions (like enchondroma, osteochondroma, osteoid osteoma) do not show significant FDG uptake [18].

## 4. Biopsy

Biopsy is often required to reach a specific diagnosis. Percutaneous image-guided biopsy is preferred over surgical open biopsy because it is less invasive, more cost effective, and has less postprocedure morbidity. This should generally be performed following discussions with the surgeon involved, respecting the regional compartment anatomy. Misadventures in terms of approach and technique can lead to significant complications, such as more radical surgery and unnecessary amputation [20]. The biopsy procedure should be performed in such a fashion that the entire biopsy tract can be excised at the time of definitive surgery [21]. It should be noted that contamination of adjacent compartments or neurovascular bundles may occur as the result of biopsy approach or procedure complications, such as hematoma [21]. Every attempt should be made to accurately stage disease, prior to performing the biopsy. If the lesion can be well visualized and accurately localized by ultrasound, we prefer this as our primary modality to help biopsy a lesion. CT is selected when the lesion is deep, predominantly osseous, and poorly visualized by ultrasound. With the emergence of vertically open magnets, it is expected that MR guided biopsies will become more popular [22].

## 5. The Influence of Age on the Differential Diagnosis of Pelvic Tumors

Many bone tumors are to a large extent age specific (Figure 8). Although, a number of cases fall outside the expected range, it is of important diagnostic significance to consider the patient's age when assessing osseous pelvic tumors.

### 5.1. Benign Tumors

 Most benign tumors occur before the age of 40.

### 5.2. Osteochondroma

**Figure 2:**
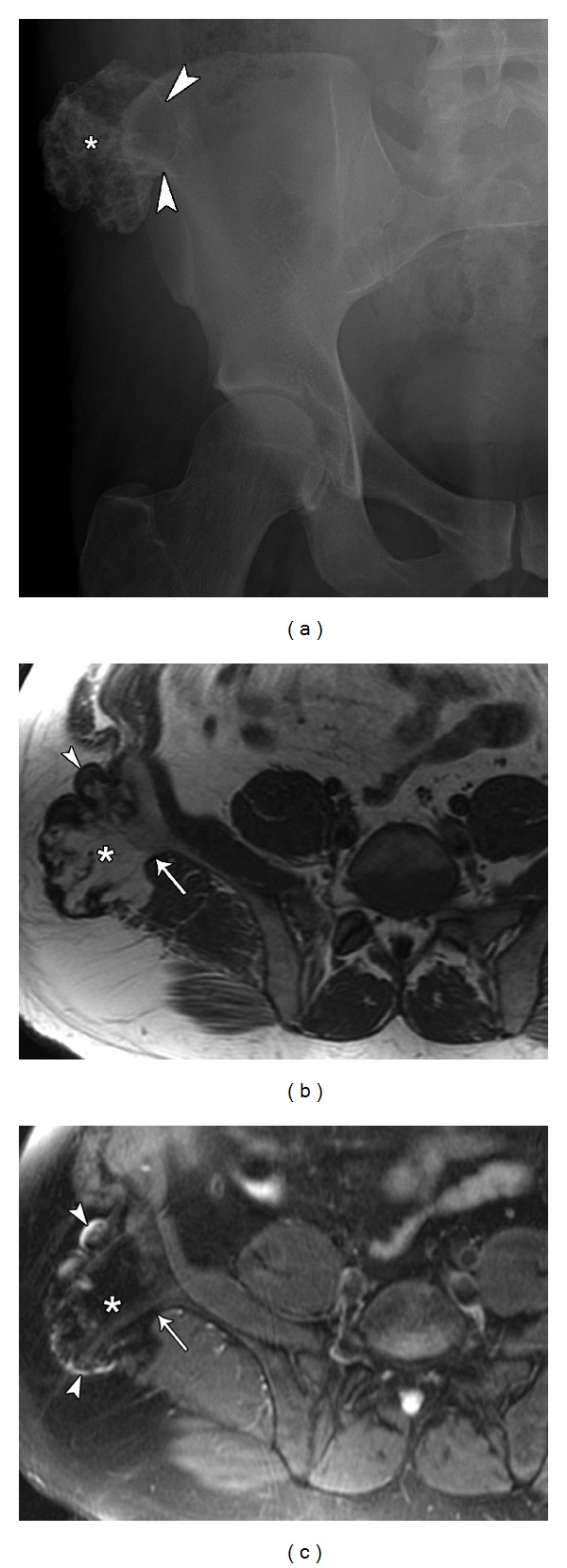
31-year-old male presents with palpable hard mass lateral to right iliac crest diagnosed as an osteochondroma. Pelvic plain film (a) demonstrates an irregularly calcified, pedunculated lesion (asterisk) arising from the right iliac crest. It presents as a peripheral outgrowth with its cortex in continuity with the iliac bone (arrowheads). Axial T1 (b) and axial T2 fat sat. (c) MRI images of same lesion (asterisk) demonstrate the continuity of the cortex and medullary portion of the lesion with the parent bone (arrow) and identify a thin cartilage cap (arrowheads).

Osteochondroma, one of the most common benign lesions of bone, is a cartilage-covered bony projection (exostosis), usually pointing away from the nearby joint. The innominate bone is involved in 5% of cases [23]. The radiographic presentation is quite characteristic, presenting either as a pedunculated lesion with a slender pedicle directed away from the growth plate, or less commonly, a sessile growth with a broad base. A distinguishing feature of this lesion is continuity of the cortex and medullary portion of the lesion with the parent bone (Figure 2). The typical clinical presentation is that of a nontender, painless mass in a younger patient. There is no major variation in size after skeletal maturity. Symptoms may be secondary in nature, either due to compression of adjacent structures, such as a nerve, or due to inflammatory changes involving the adventitious bursa covering the cartilaginous cap. In the pelvis, osteochondromas may be large and lead to soft tissue displacement. Osteochondromas, depending upon their anatomic location, may be more prone to trauma-induced fracture.

In the absence of trauma, bursitis, or nerve compression, the onset of pain in a previously asymptomatic osteochondroma could indicate malignant transformation. The extent of cartilage cap calcification is variable but must be closely evaluated in the symptomatic exostosis. A small, well-defined cap with stippled calcification is most likely benign; whereas a large, poorly defined cap, containing irregular or incomplete calcification, must be considered as a possible malignancy. Any increase in the thickness of the cartilage cap after puberty should raise concern for malignant change. A cartilage cap more than 2.0 cm thick is suspicious for malignant transformation [23, 24]. The patterns of calcification are variable and may be irregular; thus, discerning benign from a malignantly transformed lesion may be difficult.

### 5.3. Osteoblastoma

**Figure 3:**
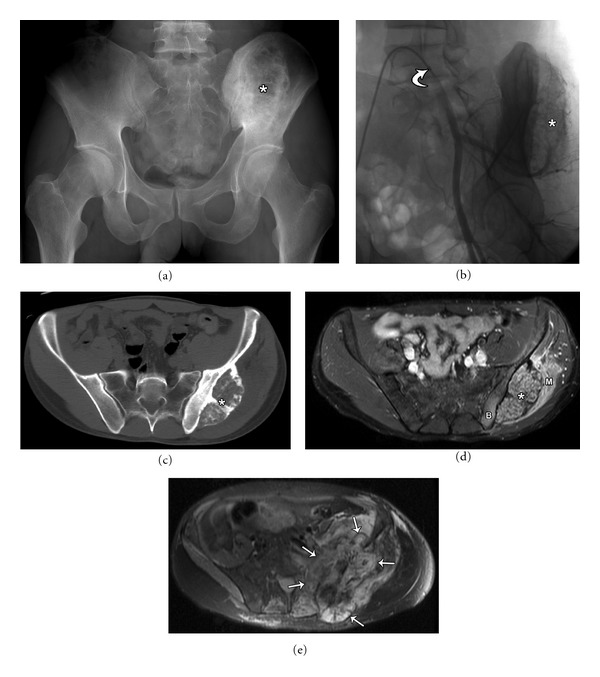
50-year-old male with osteoblastoma (aka giant osteoid osteoma) involving the left iliac bone. Plain radiograph (a) shows a large lytic left iliac bone lesion (asterisk) with sharp sclerotic borders. An angiographic image (b) with a 5 French cobra catheter (curved arrow) in the left common iliac artery demonstrates the vascular nature of the tumor, with the tumor blush (asterisk) predominantly supplied by the superior gluteal artery which was subsequently embolised a day prior to surgical resection. The axial CT (c) and axial T2 MRI (d) demonstrates the mass originating from the outer cortex of the right iliac bone. It has a narrow zone of transition with distinct borders. Edema is noted in the iliac bone (B) and the gluteal musculature (M) on the fluid sensitive T2 MRI sequence demonstrating the aggressive nature of this benign lesion. Axial T2 MRI (e) 5 years postresection demonstrates unusual progression to high grade osteoblastic osteosarcoma (arrows). Even though benign, osteoblastomas can be locally aggressive, recur or differentiate to a malignant aggressive tumor, in this case necessitating a revision hemipelvectomy.

These lesions occur most commonly in the second and third decades of life and are approximately two times more common in men than women [25, 26]. Unfortunately, the radiographic appearances of osteoblastoma are varied and generally nonspecific (Figure 3(a)). They range from being osteolytic to osteosclerotic and a spectrum of combinations in between. The characteristic lesion consists of a geographic pattern of lysis, with or without a rim of surrounding sclerosis [24, 25]. Osseous expansion, cortical thinning, and a soft tissue mass may be present, mimicking a more aggressive process (Figures 3(b), 3(c), and 3(d)). When radiographs demonstrate an expansile, well-circumscribed, partially calcified lesion, the diagnosis of osteoblastoma should be considered. Bone sclerosis and an exuberant periostitis are well described, often raising concern for malignancy. Despite the benign nature of “conventional” osteoblastoma, an inflammatory response within the adjacent bone marrow and soft tissues may lead to an aggressive appearance on MRI (Figure 3(d)) and can recur [27]. Osteoblastomas may demonstrate secondary aneurysmal bone cyst formation, similar to giant cell tumor; however, unlike giant cell tumor, the solid portion of osteoblastoma is characteristically high signal on T2-weighted images [28].

### 5.4. Giant Cell Tumor

**Figure 4:**
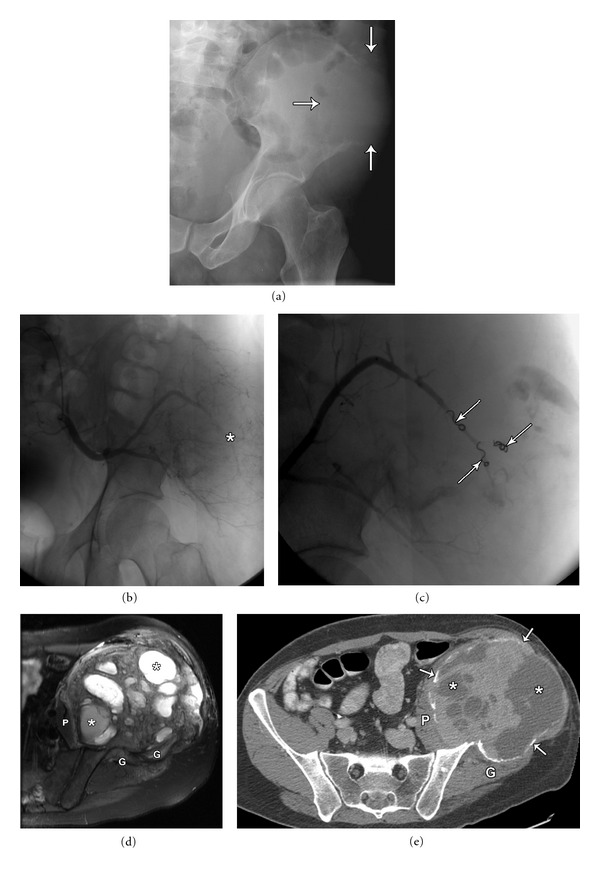
40-year-old male with a large left iliac wing expansile mass diagnosed as pelvic giant cell tumor. Plain film (a) shows a large, lytic, destructive lesion in the right iliac wing with indistinct margins and cortical breakthrough (arrows), with no visible matrix. Left internal iliac artery angiogram (b) demonstrating the highly vascular nature of the tumor. Selective embolization of the superior gluteal artery (c) demonstrating embolization coils (arrowheads), and decreased tumor vascularity. Axial T2 fat sat MRI (d) and axial post-contrast CT images (e) demonstrating expansile nature of the giant cell tumor with multiple foci of necrosis (asterisk) and intervening tumor stroma. Size of the mass increased following embolization due to internal bleeding and necrosis. Displacement of surrounding soft tissues (Iliopsoas (P), gluteal musculature (G)), rather than invasion is illustrated. Note residual outer rim of expansile cortical bone (arrows), better appreciated on the CT scan.

Giant cell tumors (GCTs) have peak prevalence in the third and fourth decades of life and are slightly more common in women than men [25, 29, 30]. Only 1% to 3% occurs in skeletally immature individuals [29] with 98% of cases, occurring after physeal plate closure [31]. GCT can show locally aggressive features and are very vascular in nature. Approximately 5%–10% of giant cell tumors are malignant [32]. It invariably involves the epiphysis but is centered in the metaphysis. GCT in the innominate bone favors the epiphyseal equivalent, that is, adjacent to the sacroiliac joint or hip articulation [33]. Radiographic appearances of GCT are highly characteristic in long bones, whereas those seen in flat bone are less specific. The sacrum is the commonest site of occurrence in the spine (4%) [34]. Pelvic GCT is generally lytic and the large associated soft tissue mass may resemble an aggressive lesion demonstrating increased vascularity (Figure 4(a)). Occasionally, sacral lesions will cross the sacroiliac joint. The absence of internal punctate calcification, intralesional bone formation, and significant periostitis, unless complicated by fracture, is helpful in GCT diagnosis [29, 30]. GCT often demonstrates solid and cystic components, leading to the radiological appearance of secondary aneurysmal bone cyst formation (Figures 4(d), and 4(e)). Fluid/fluid levels may be identified but are not specific to GCT [31]. The primary role of MRI is to define intraosseous, intraarticular and soft tissue involvement (Figure 4(d)). The lesion typically demonstrates low T1 and heterogeneous T2 signal intensity. Of note, the solid component has a characteristic appearance; demonstrating low signal on both T1- and T2-weighted images. This low-to-intermediate signal intensity in the solid portion of the tumor has been considered secondary to the presence of hemosiderin, high collagen content, or high cellularity [29, 30]. Inhomogeneity of internal signal reflects necrosis and cystic change. Haemosiderin changes may present, from previous hemorrhage.

### 5.5. Fibrous Dysplasia

**Figure 5:**
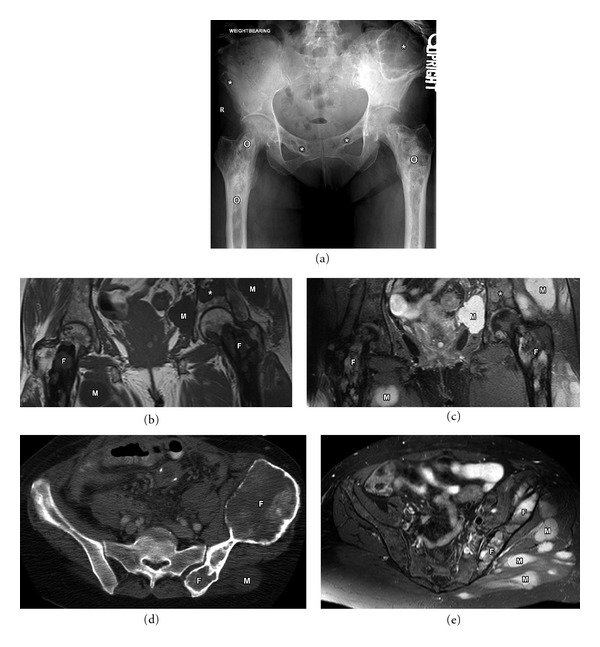
61-year-old female with Mazabraud syndrome (polyostotic fibrous dysplasia and soft tissue myxomas (M)). Plain film (a) demonstrates multiple lucent lesions with ground glass matrix in the pelvis (asterisk) and long predominantly sclerotic abnormality in bilateral femurs (circle). Fibrous dysplasia is often described as a long lesion in the long bone. Coronal T1 (b) and STIR (c) MRI images demonstrating multiple fibrous dysplasia (F) lesions. Note that some of these maintain low to intermediate signal intensity on both T1 and T2 images (asterisk). Axial CT (d) and axial T2 fat sat (e) MRI images demonstrating contour abnormality of the left ilium with ground glass matrix and focal bone expansion (F). Multiple T2 bright myxomas (M) are noted in the gluteal musculature.

Fibrous dysplasia is a benign developmental abnormality of bones, of unknown cause, in which there is failure of normal ossification, deposition of fibrous tissue and modulation deformity. Two broad categories are described: monostotic and polyostotic. Monostotic fibrous dysplasia is more common than the polyostotic form, and accounts for 75 to 80% of cases [35]. Polyostotic type is, however, much more common in two syndromes. In Mazabraud syndrome, fibrous dysplasia is associated with soft tissue myxomas, lesions which are predominantly intramuscular in nature (Figure 5). In McCune Albright syndrome, polyostotic fibrous dysplasia is associated with sexual precocity and skin pigmentation. Most cases of fibrous dysplasia are asymptomatic, so true incidence is unknown. Unfortunately, there are very few defining features of the disease. This entity can be seen in a patient of any age and the radiographic appearances vary. In the pelvis, typical fibrous dysplasia lesions are lytic and particularly occur in the iliac bones (Figure 5(a)). There is a classical description of a “ground glass” matrix, and patchy sclerosis, as it resolves. It is very unusual to observe a periosteal reaction. The signal intensity characteristics of fibrous dysplasia vary and are nonspecific. When associated with secondary bone cyst, they can demonstrate fluid-fluid levels [36]. Typically, lesions tend to have low signal intensity on T1- and T2-weighted sequences (Figures 5(b), and 5(c)); however, they may be of higher signal intensity on T2 [6].

### 5.6. Aneurysmal Bone Cyst

**Figure 6:**
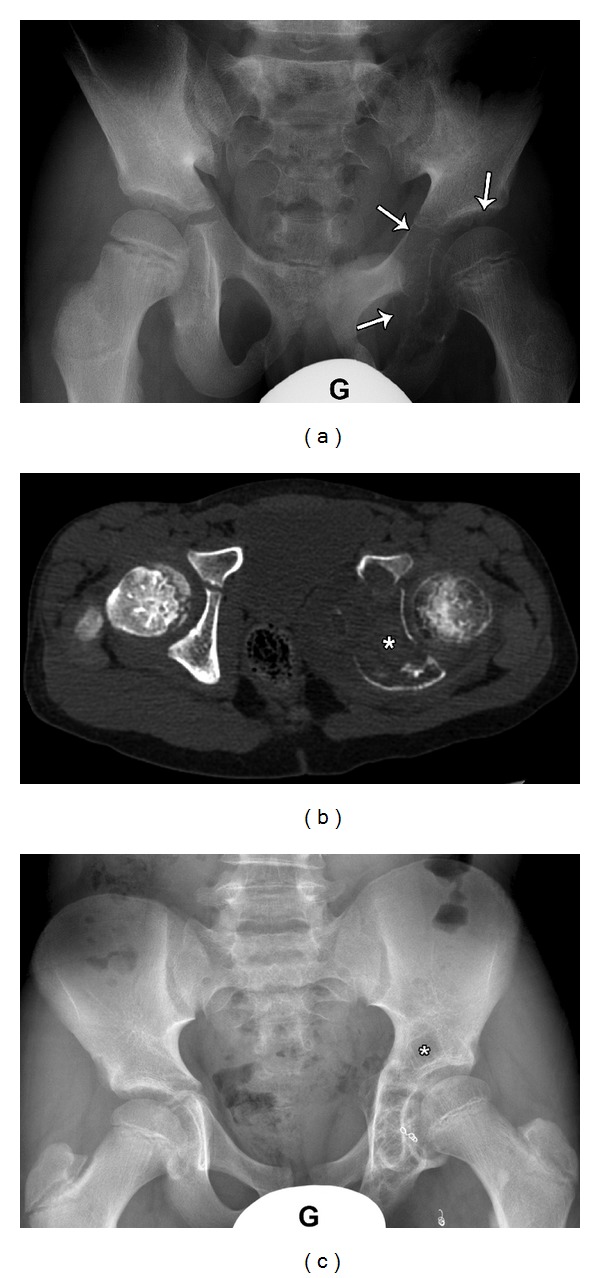
8-year-old male presented with left hip pain, diagnosed as Aneurysmal bone cyst. Plain film (a) demonstrates large expansile lytic lesion (arrows) involving medial left acetabulum extending to the inferior pubic rami (G: gonadal shield). Axial CT scan (b) obtained immediately following curettage and packing with bone allograft showing relatively dense material (asterisk) within the expanded left Ischium. Follow-up plain film (c) five years postsurgical curettage and embolization (note the embolization coils along the medial aspect of the hip) shows healing of the ABS with return to normal contour and course trabeculation. Unfortunately, a focus of recurrence is also noted in the superior acetabulum (asterisk). Local recurrence is not common, but possible.

The World Health Organization defines aneurysmal bone cyst (ABC) as an expansile osteolytic lesion (Figure 6) with blood-filled spaces separated by connective tissue septa containing trabeculae of bone or osteoid, as well as osteoclast giant cells [37]. These lesions are most common in patients less then 20 years of age but can be seen in older patients. These lesions are slightly more common in females than males [38]. Fifty percent of flat bone ABCs are found in the pelvis [39].

The origin of ABCs is not well understood. Authors in the past have suggested these lesions may develop *de novo* in bone or maybe secondary to hemorrhagic degeneration of a preexisting lesion such as a giant cell tumor, hemangioma, osteoblastoma, or fibrous dysplasia; however, elements definitive of other osseous lesions are not usually found in pathological specimens, and as such, the etiology of ABC remains controversial [40, 41].

Lesions are typically eccentric and lytic, with well-circumscribed borders. The overlying cortex is expansile and thinned (Figure 6(b)). A sclerotic rim and internal trabeculation may be present. Computed tomography and magnetic resonance imaging may show cystic spaces with fluid-fluid levels and contrast enhancing internal septa. High-to-low T1- and-T2 weighted signal intensity in the fluid-fluid levels is presumably due to blood products of varying age. On MRI, edema may be present in the surrounding bone [40].

Needle biopsy can be attempted for diagnosis; however, the specimens may only yield blood products and can be nondiagnostic. Open biopsy with frozen section is more likely to provide definitive diagnosis [41].

### 5.7. Chondroblastoma

**Figure 7:**
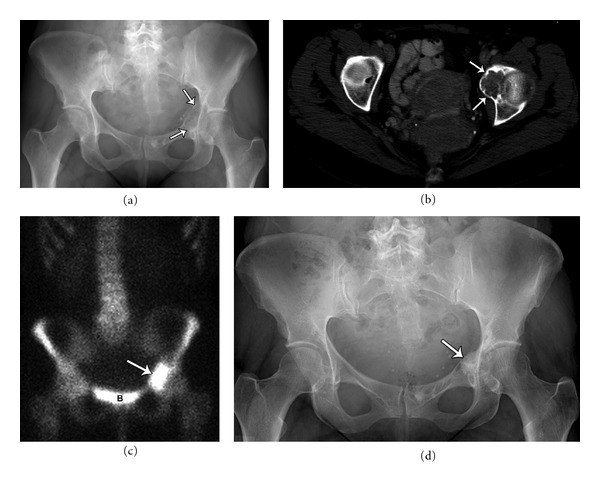
Forty-six-year-old female with a lytic lesion in the left medial acetabulum, diagnosed as chondroblastoma. Plain film (a) and axial CT (b) demonstrating a lytic lesion with heterogeneous chondroid matrix and sharp scalloped margins. The tumor showed increased uptake on bone scan (c) (B: bladder). Plain film (d) demonstrating healed chondroblastoma following surgical curettage and cement packing.

**Figure 8:**
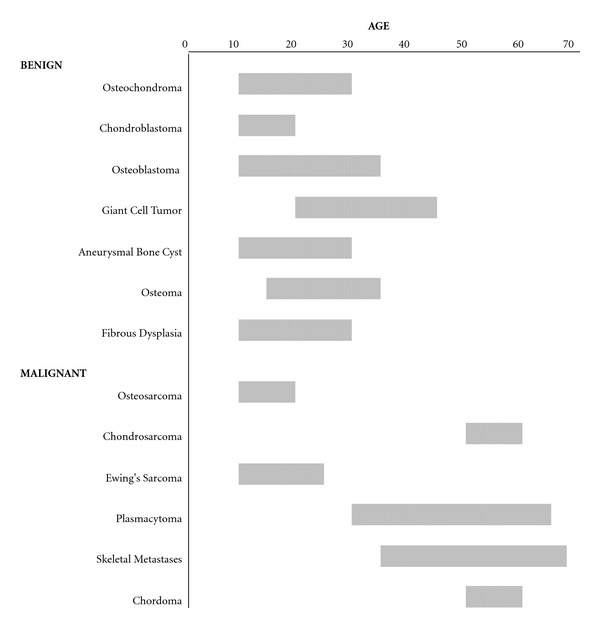
Typical ages for benign and malignant osseous pelvic tumors.

A Chondroblastoma is considered a benign cartilage tumor originating in the epiphysis, often with an aggressive appearance. It usually presents clinically with pain in the 2nd decade of life. It typically appears as an expansile lytic lesion with a faint chondroid matrix, scalloped borders, and an intact but thinned cortex (Figures 7(a), and 7(b)). It characteristically has extensive bone marrow edema on MRI and can show increased uptake on bone scan (Figure 7(c)). It can demonstrate fluid-fluid levels. The femoral head is a more preferred location for chondroblastoma than the pelvis. These tumors are normally treated by curettage and packing (Figure 7(d)). There is a high risk of local recurrence and on rare occasions, there have been pulmonary metastasis.

## 6. Summary

We have discussed and illustrated common benign tumors that affect the skeletal pelvis. Although there is a wide differential diagnosis for skeletal pelvic tumors, proper use of plain radiography, CT, and MR imaging can help narrow the diagnostic considerations and clearly define the anatomical extent. Biopsy may ultimately be necessary for final diagnosis.
